# An Isoflavone from *Dipteryx alata* Vogel is Active against the *in Vitro* Neuromuscular Paralysis of *Bothrops jararacussu* Snake Venom and Bothropstoxin I, and Prevents Venom-Induced Myonecrosis

**DOI:** 10.3390/molecules19055790

**Published:** 2014-05-06

**Authors:** Miriéle C. Ferraz, Edson H. Yoshida, Renata V.S. Tavares, José C. Cogo, Adélia C.O. Cintra, Cháriston A. Dal Belo, Luiz M. Franco, Márcio G. dos Santos, Flávia A. Resende, Eliana A. Varanda, Stephen Hyslop, Pilar Puebla, Arturo San Feliciano, Yoko Oshima-Franco

**Affiliations:** 1 Post-Graduate Program in Pharmaceutical Sciences, University of Sorocaba (UNISO), Rodovia Raposo Tavares, Km 92.5, 18023-000 Sorocaba, SP, Brazil; E-Mails: miriele.ferraz@gmail.com (M.C.F.); edsonyoshida@bol.com.br (E.H.Y.); 2 Post-Graduate Program in Technological and Environmental Processes, University of Sorocaba (UNISO), Rodovia Raposo Tavares, Km 92.5, 18023-000 Sorocaba, SP, Brazil; E-Mail: renavasques@yahoo.com.br; 3 Serpentarium of the Vale do Paraíba University (CEN—UNIVAP), Av Shishima Hifumi 2911, 12244-000 São José dos Campos, SP, Brazil; E-Mail: jccogo@univap.br; 4 Department of Clinical, Toxicological and Bromatological Analysis, Faculty of Pharmaceutical Sciences, São Paulo University (USP), Via do Café S/N, 14040-903 Ribeirão Preto, SP, Brazil; E-Mail: acocintra@hotmail.com; 5 LANETOX, Federal University of Pampa (UNIPAMPA), Avenida Antonio Trilha 1847, 97300-000 São Gabriel, RS, Brazil; E-Mail: charistonbelo@unipampa.edu.br; 6 Methodist University of Piracicaba, Rodovia do Açucar, Km 156, 13423-170 Piracicaba, SP, Brazil; E-Mail: lenof@terra.com.br; 7 Post-Graduate Course in Environmental Sciences, Federal University of Tocantins (UFT), Av NS 15 ALC NO 14, 109 Norte, 77001-090 Palmas, TO, Brazil; E-Mail: galdino@uft.edu.br; 8 Faculty of Pharmaceutical Sciences, São Paulo State University (UNESP), Rodovia Araraquara-Jau, Km 1, 14801-902 Araraquara, SP, Brazil; E-Mails: flaviabiomed@yahoo.com.br (F.A.R.); eavaranda@gmail.com (E.A.V.); 9 Department of Pharmacology, Faculty of Medical Sciences, State University of Campinas (UNICAMP), Rua Tessália Vieira de Camargo, 126, 13083-887 Campinas, SP, Brazil; E-Mail: hyslop@fcm.unicamp.br; 10 Department of Pharmaceutical Chemistry, Salamanca University, CIETUS, IBSAL, Salamanca 37007, Spain; E-Mails: puebla@usal.es (P.P.); asf@usal.es (A.S.F.)

**Keywords:** ames test, bothropstoxin-I, 7,8,3'-trihydroxy-4'-methoxyisoflavone, neuromuscular junction, *Salmonella* mutagenicity, snake venoms

## Abstract

Snakebite is a neglected disease and serious health problem in Brazil, with most bites being caused by snakes of the genus *Bothrops*. Although serum therapy is the primary treatment for systemic envenomation, it is generally ineffective in neutralizing the local effects of these venoms. In this work, we examined the ability of 7,8,3'-trihydroxy-4'-methoxyisoflavone (TM), an isoflavone from *Dipteryx alata*, to neutralize the neurotoxicity (in mouse phrenic nerve-diaphragm preparations) and myotoxicity (assessed by light microscopy) of *Bothrops jararacussu* snake venom *in vitro*. The toxicity of TM was assessed using the *Salmonella* microsome assay (Ames test). Incubation with TM alone (200 μg/mL) did not alter the muscle twitch tension whereas incubation with venom (40 μg/mL) caused irreversible paralysis. Preincubation of TM (200 μg/mL) with venom attenuated the venom-induced neuromuscular blockade by 84% ± 5% (mean ± SEM; *n* = 4). The neuromuscular blockade caused by bothropstoxin-I (BthTX-I), the major myotoxic PLA_2_ of this venom, was also attenuated by TM. Histological analysis of diaphragm muscle incubated with TM showed that most fibers were preserved (only 9.2% ± 1.7% were damaged; *n* = 4) compared to venom alone (50.3% ± 5.4% of fibers damaged; *n* = 3), and preincubation of TM with venom significantly attenuated the venom-induced damage (only 17% ± 3.4% of fibers damaged; *n* = 3; *p* < 0.05 compared to venom alone). TM showed no mutagenicity in the Ames test using *Salmonella* strains TA98 and TA97a with (+S9) and without (−S9) metabolic activation. These findings indicate that TM is a potentially useful compound for antagonizing the neuromuscular effects (neurotoxicity and myotoxicity) of *B. jararacussu* venom.

## 1. Introduction

Envenomation by *Bothrops* snakes is characterized by local (pain, edema, inflammation, blistering, hemorrhage and necrosis) and systemic (coagulopathy, systemic hemorrhage, acute kidney injury and circulatory shock) manifestations [1]. *Bothrops jararacussu* is a large pit viper found in southeastern Brazil and northern Argentina [2]. Envenomation by this species shares many of the foregoing features with other *Bothrops* species [3], with most of the clinical manifestations of envenoming being mediated predominantly by snake venom metalloproteases (SVMPs), serine proteases, phospholipases (PLA_2_) and C-type lectins. Transcriptomic analysis has confirmed that these are indeed the major protein classes in this venom, with PLA_2_ being particularly abundant: Lys49-PLA_2_ homologs accounted for 83.2% of PLA_2_ transcripts, acidic Asp49-for 0.6% and basic Asp49-PLA_2_ for 0.1% [4].

In addition to the features indicated above, clinical studies in the early 1900s suggested that bites by *B. jararacussu* also involved systemic manifestations reminiscent of envenoming by the South American rattlesnake, *Crotalus durissus terrificus*, viz., neurotoxicity, blindness, blurred vision, difficulty in swallowing and paralysis [5], in addition to other unspecified signs of neurotoxicity [6,7,8]. In agreement with this, various studies using isolated vertebrate nerve-muscle preparations *in vitro* have shown that *B. jararacussu* venom [9,10] and PLA_2_ from this venom [11,12,13,14,15,16] can cause neuromuscular blockade by pre- and post-synaptic mechanisms.

Serum therapy, the treatment of choice for snakebites, efficiently neutralizes the systemic manifestations but is generally ineffective against the local effects (edema, inflammation, hemorrhage and necrosis) of venoms [17]. Extensive local tissue damage leading to tissue loss after *Bothrops* bites can result in permanent disability and amputations [18,19]. Hence, there is a clinical and therapeutic impetus to develop alternatives for treating these local manifestations, with plant-derived bioactive products providing important candidate or lead molecules [20,21].

Several studies have shown that the neurotoxic and myotoxic effects of *B. jararacussu* venom and its PLA_2_ myotoxins can be neutralized by some plant extracts and their isolated compounds [21,22,23,24,25,26,27,28,29,30]. In particular, a methanolic extract of bark from *Dipteryx alata* Vogel, a plant species native to the Brazilian cerrado, fully protects against the neuromuscular blockade caused by *B. jararacussu* venom, whereas a dichloromethane extract provides partial protection against the blockade caused by *B. jararacussu* and *C. d. terrificus* venoms [31]. Lupane triterpenoids (lupeol, lupenone, betulin and 28-OH-lupenone) from *D. alata* prevent the paralysis induced by *B. jararacussu* venom [32], whereas betulin and lupenone only partially neutralize the paralysis induced by *C. d. terrificus* venom [33].

To increase our understanding of the components involved in the protective action of *D. alata* extracts, in this study we examined the ability of 7,8,3'-trihydroxy-4'-methoxyisoflavone (TM), an isoflavone from *D. alata* [32], to attenuate the neurotoxicity and myotoxicity of *B. jararacussu* venom and its main myotoxin, bothropstoxin-I (BthTX-I), in mouse phrenic nerve-diaphragm preparations *in vitro*. The mutagenicity (toxicity) of TM, as an indicator of it potential use as a clinical agent, was assessed in the Ames test using *Salmonella* strains TA 98 and TA 97a.

## 2. Results and Discussion

This work was based on three premises: (1) an understanding of the principal clinical manifestations of *B. jararacussu* envenomation [1]; (2) the low efficacy of antivenom against the local effects of *Bothrops* venoms [18,19]; and (3) the availability of a molecule, 7,8,3'-trihydroxy-4'-methoxyisoflavone (TM) from *D. alata*, with potentially interesting activity against *B. jararacussu* snake venom.

### 2.1. Molecule

**Figure 1:**
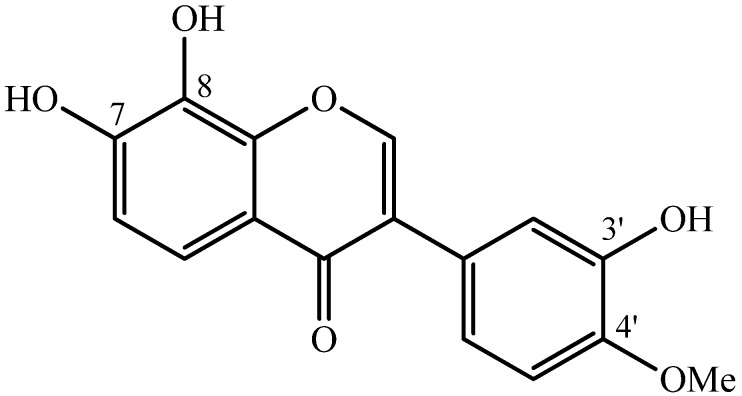
Chemical structure of 7,8,3'-trihydroxy-4'-methoxyisoflavone isolated from *D. alata* Vogel [32].

Figure 1 shows the chemical structure of TM from *D. alata* [32], a compound also found in *Xanthocercis zambesiaca* (Baker) plant extract [34].

In preliminary screening for anti-snake venom activity, fraction F7 of a dichloromethane extract of *D. alata* bark [32] showed the best activity out of 11 fractions when tested against the neuromuscular activity of *B. jararacussu* venom [31]. Isoflavonoids are a large and very distinctive subclass of flavonoids, but only a few plants, including *D. alata*, have been reported to contain isoflavonoids [35]. Isoflavones are biologically active plant-food constituents that are common in the human diet and food industry, and are widely used as preservatives in pharmaceutical products [36]. This range of activities and uses suggested the possibility that isoflavones could be potentially useful as an adjuvant treatment for snakebites alongside standard serum therapy, particularly since antiserum shows little or no neutralizing activity towards the local effects of *Bothrops* venoms [18,19].

### 2.2. Pharmacological Assays

**Figure 2:**
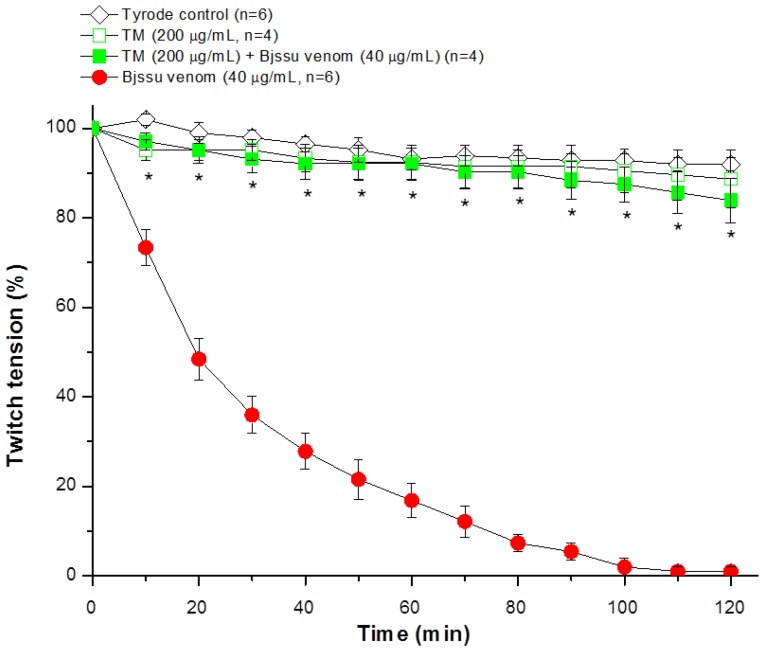
Twitch tension responses of indirectly stimulated PND incubated with 7,8,3'-trihydroxy-4'-methoxyisoflavone (TM, 200 μg/mL), *B. jararacussu* (Bjssu) venom (40 μg/mL) or a mixture of TM (200 µg/mL) and venom (40 μg/mL). The TM + venom mixture was preincubated at 37 °C for 30 min prior to testing. The points are the mean ± SEM of the number of experiments indicated in the figure. * *p* < 0.05 for TM + venom compared to venom alone.

The ability of TM to inhibit the neuromuscular blockade caused by *B. jararacussu* venom was investigated in mouse phrenic nerve-diaphragm (PND) preparations. Figure 2 shows the characteristic irreversible neuromuscular blockade induced by *B. jararacussu* venom *in vitro* (Bjssu, 40 µg/mL, *n* = 6, * *p* < 0.05 compared to control), in agreement with that originally reported by Rodrigues-Simioni *et al.* [9]. Incubation with TM alone (200 µg/mL, *n* = 4) did not change the muscle twitch-tension responses, which were similar to those of preparations incubated with Tyrode solution (negative control; *n* = 6). Preincubation of TM (200 µg/mL) with venom (40 µg/mL) for 30 min prior to testing totally abolished the venom-induced neuromuscular blockade (*n* = 4, * *p* < 0.05 compared to venom alone).

**Figure 3:**
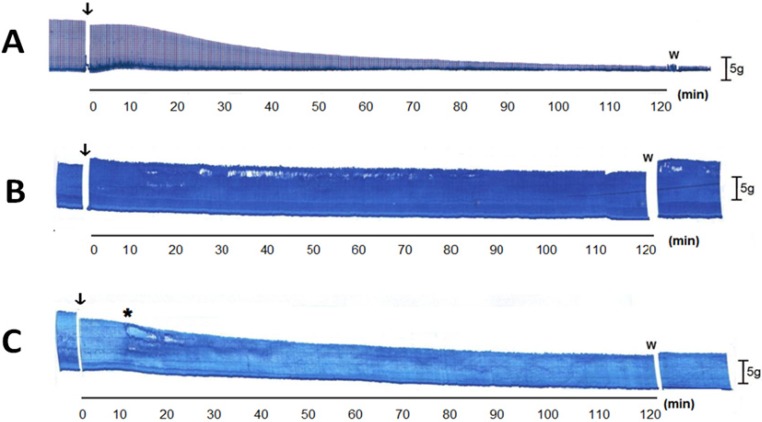
Neuromuscular responses of indirectly stimulated PND incubated with BthTX-I (20 µg/mL) in the absence (**A**) and presence (**B**,**C**) of 7,8,3'-trihydroxy-4'-methoxyisoflavone (TM, 200 µg/mL). In (**B**), TM (200 µg/mL) was preincubated with BthTX-I (20 µg/mL) at 37 °C for 30 min before addition to the bath, whereas in (**C**) TM (200 µg/mL) was added separately (at *) 10 min after the addition of BthTX-I (20 µg/mL). Arrows show the moment of sample addition to the bath. Bar = tension of 5 g/cm; W = wash.

*Bothrops jararacussu* venom is rich in PLA_2_, most of which are Lys49-PLA_2_, with considerably fewer basic and acidic Asp49-PLA_2_ [4]. Bothropstoxin-I (BthTX-I) is the major Lys49-PLA_2_ myotoxin and totally reproduces the *in vitro* neurotoxicity and myotoxicity of *B. jararacussu* venom [11]. This toxin acts presynaptically before causing membrane depolarization [15,37]. As shown in Figure 3A, incubation of PND preparations with BthTX-I (20 µg/mL) mimicked the characteristic progressive, irreversible decrease in muscle twitch-tension caused by the venom. As with the venom, preincubation of BthTX-I (20 µg/mL) with TM (200 µg/mL) fully protected the PND against toxin-induced neuromuscular blockade (Figure 3B). When TM was added 10 min after BthTX-I there was still attenuation of the neuromuscular blockade, although this was much less marked than with the preincubation protocol (Figure 3C). These findings suggest that the protective action of TM against venom-induced neuromuscular blockade most likely resulted from the inhibition of BthTX-I, presumably by attenuating the presynaptic activity of the toxin [15,37].

### 2.3. Quantitative Histological Analysis

**Figure 4:**
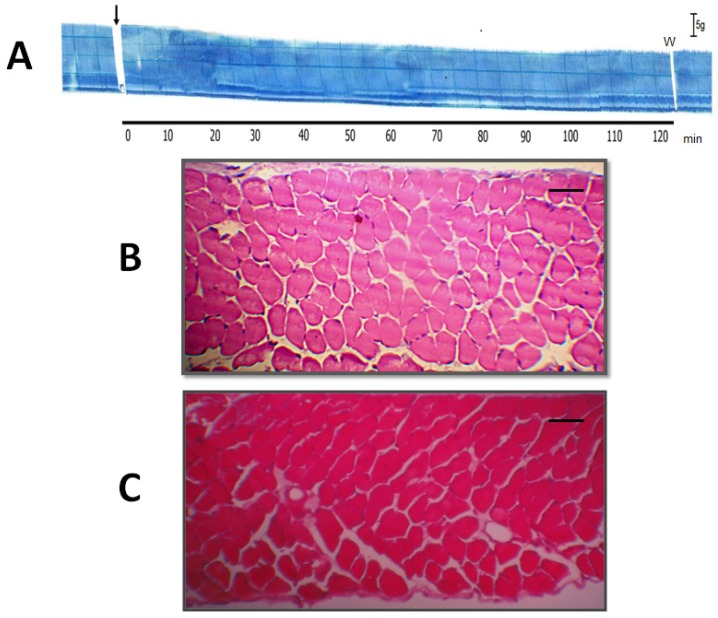
Histological analysis of muscle damage in PND incubated with or without 7,8,3'-trihydroxy-4'-methoxyisoflavone (TM). Panel (**A**) shows a representative trace of PND incubated with TM (*n* = 4). The arrow indicates the addition of TM. Bar = tension of 5 g/cm. W = wash. Panels B and C show cross-sections of diaphragm muscle incubated with Tyrode solution alone (negative control; *n* = 6) (**B**) or TM alone (*n* = 4) (**C**). Note the normal appearance of the fibers (polygonal aspect and peripheral nuclei) in both panels. Bar = 50 μm in B and C.

To assess the anti-myotoxic activity of TM, diaphragm muscles exposed to different treatments (TM, venom or TM + venom) were analyzed by light microscopy, as described by Queiroz *et al.* [38] for mouse tibialis anterior muscle. Figure 4A shows a characteristic myographic recording of PND contractile activity during incubation with TM (200 µg/mL) for 120 min; similar responses were observed in preparations incubated with Tyrode solution alone (results not shown). The percentage of damage cells in these two protocols was <10% [9.7% ± 1.6% (*n* = 6) for Tyrode solution and 9.2% ± 1.7% (*n* = 4) for TM-treated muscles].

**Figure 5:**
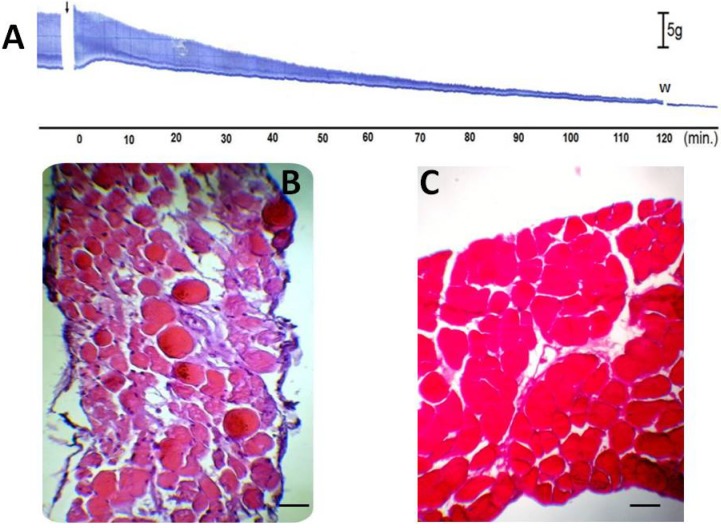
Neuromuscular responses of indirectly stimulated PND incubated with *B. jararacussu* venom (40 μg/mL) for 120 min (**A**). Notice the irreversible blockade. Venom was added at the arrow. Bar = tension of 5 g/cm. W = wash. Panel (**B**)—Cross-section of diaphragm muscle incubated with venom alone (40 μg/mL, *n* = 6). Note the edema and intense myonecrosis (muscle fiber atrophy, hyaline aspect, sarcolemmal disruption and myofibril lysis). Panel (**C**)—Cross-section of diaphragm muscle incubated with a mixture of TM (200 μg/mL) + venom (40 μg/mL). Note the normal appearance of the fibers (polygonal aspect and peripheral nuclei), but with occasional edema. Bar = 50 μm for B and C.

In indirectly stimulated PND, *B. jararacussu* venom (40 μg/mL, *n* = 6) induced a transient contracture that was followed by progressive neuromuscular blockade of muscle twitches during the following 120 min (Figure 5A). In contrast to the foregoing controls, there was a marked increase in the percentage of damaged fibers (to 50.3% ± 5.4%; *p* < 0.05 compared to the Tyrode control) in diaphragms incubated with venom alone (40 μg/mL, *n* = 3) for 120 min (Figure 5B). In diaphragm muscle incubated with a mixture of TM (200 μg/mL) + venom (40 μg/mL) (Figure 5C, *n* = 3) there was a significant decrease in the percentage of damaged fibers (17% ± 3.4%; *p* < 0.05 compared to venom alone). Although the venom produced complete neuromuscular blockade, quantitative analysis of the fibers showed that only 50% were damaged. This discrepancy may reflect the fact that muscle cross-sections do not allow observation of the entire fiber length. Although diaphragm muscle has a high resistance to fatigue and a safety margin of 10% [39], the venom nevertheless had a striking effect on the physiological excitation-contraction coupling mechanism. As shown here, the preincubation of venom with TM was effective in protecting the muscle against venom-induced myotoxicity.

In this work, we focused on the ability of TM to antagonize the neuromuscular action (neurotoxicity and myotoxicity) of *B. jararacussu* venom and its main PLA_2_ myotoxin, BthTX-I. However, *B. jararacussu* venom has a variety of other biological activities (edema-formation, hemorrhage and lethality) against which it would be interesting to assess the neutralizing capacity of TM. Nevertheless, with the isolation procedure used in this study, the amount of isoflavone obtained was low, which severely limited the number of protocols and activities that could be tested. This limitation (low yield/availability of purified TM) is common to other phytochemical compounds with biological activity [40]. Nonetheless, as shown here, TM faithfully mimicked the protection against the venom-induced neuromuscular blockade seen with a crude extract of *D. alata* [31].

Several mechanisms have been proposed to explain the anti-snake venom activity of phytochemical compounds [21], with the specific interactions depending on the plant compound (tannins, coumarins, flavonoids, *etc.*) and the snake genus involved. In the case of TM, we suggest that its interaction with *B. jararacussu* venom and BthTX-I involves the hydroxyl groups at positions C-8 and C-7 of the isoflavone (see Figure 1). Since the neighboring hydroxyls are susceptible to radical formation [35], this chemical interaction would ultimately lead to a very reactive 7,8-quinone moiety that could modify specific sites in enzymes responsible for the local and systemic effects of the venom.

### 2.4. Salmonella Mutagenicity Assay

The toxicology of TM was assessed in the *Salmonella* mutagenicity assay, using test strains TA97a and TA98 that can be reverted by frameshift mutagens [41,42]. Although the Organisation for Economic Cooperation and Development (OECD) [41] recommends the use of at least five *Salmonella* strains to meet regulatory demands, the low yield of isoflavonoid limited the use of five strains in this work. On the other hand, satisfactory toxicological results have been obtained using only TA97a and TA98 [43] or TA97a and TA100 [44], and were considered as valid mutagenic screening. In our experimental conditions a GC frameshift was evaluated. The mutagenicity assays showed that TM did not increase the number of revertant colonies relative to the negative control, indicating no mutagenicity for this compound (Table 1).

**Table 1:** Assessment of the mutagenicity of 7,8,3'-trihydroxy-4'-methoxyisoflavone (TM) against *S. typhimurium*. The table shows the revertants per plate, the standard deviation and the mutagenicity index (in brackets) for strains TA98 and TA97a after treatment with TM.

TM				
Treatment	TA 98		TA 97a	
mg/plate	−S9	+S9	−S9	+S9
DMSO	20 ± 2	30 ± 2	151 ± 8	143 ± 8
0.19	20 ± 4 (1.0)	34 ± 3 (1.1)	194 ± 4 (1.3)	172 ± 4 (1.2)
Control+	1319 ± 41 ^a^	1696 ± 41 ^b^	1875 ± 62 ^a^	1623 ± 48 ^b^

The values are the mean ± SEM of two determinations. DMSO: dimethyl sulfoxide (50 μL/plate; negative control); Control+: positive control; ^a^ 4-nitro-*o*-phenylenediamine (10 μg/plate); ^b^ 2-anthramine (1.25 μg/plate).

The genes affected in *Salmonella* strains TA98 and TA97a were *his*D6610 and *his*D3052, respectively, although these strains also contained the mutations ∆*urv*B, *rfa* and pKM101. The former was designed to enhance the mutagenicity of compounds, presumably through the nucleotide excision repair system [42] that extended into a gene for biotin synthesis and required the addition of biotin to the culture medium because of the deletion in this region. The *rfa* mutation changes the properties of the bacterial cell wall and results in partial loss of the lipopolysaccharide (LPS) barrier, thereby increasing the permeability of cells to certain chemicals; this mutation is generally detected based on the altered sensitivity to crystal violet. The R factor plasmid (pKM101) makes the strains more responsive to a variety of mutagens [45].

## 3. Experimental

### 3.1. Plant Material and Extraction

Bark samples were collected from an adult *D. alata* Vogel tree in Pedro Afonso (Tocantins, Brazil) in December 2007 and identified by Dr. Roseli B. Torres (Institute of Agronomy of Campinas—IAC). A voucher specimen was deposited in the herbarium of the IAC (IAC 50629). The bark (1.269 kg) was dried at 37 °C for 48 h and then powdered, ground in a mill, macerated for 5 days (200 g in 2 L of 70% ethanol) and the suspension then percolated (protected from light) at 20 drops/min, resulting in a 20% (*m*/*v*) hydroalcoholic extract. The extract was concentrated under reduced pressure and lyophilized to yield a final residue of 170 g that corresponded to an extraction efficiency of 85% [46].

#### 3.1.1. Isolation of 7,8,3'-trihydroxy-4'-methoxyisoflavone

For isolation of the isoflavone, lyophilized extract (50 g) was dissolved in a methanol/water mixture (80:20, *v*/*v*) and partitioned successively with the corresponding solvents to yield, after concentration, hexane (1.5 g), CH_2_Cl_2_ (18 g), EtOAc (3.7 g) and MeOH (21 g) residues. The CH_2_Cl_2_ fraction was subjected to silica gel flash column chromatography and eluted with hexane/EtOAc (9:1). This procedure yielded 12 subfractions that were further successively flash-chromatographed on silica gel and purified by Sephadex LH-20 column chromatography and eluted with hexane/CH_2_Cl_2_/MeOH (2:2:1) to yield 18 compounds, including 19 mg of 7,8,3'-trihydroxy-4'-methoxyisoflavone (TM), characterized [32] as a yellow amorphous solid. IR (KBr) cm^−1^: 3429, 1619, 1604, 1290, 1124. ^1^H-NMR (CD_3_OD) δ: 3.80 (s, 3H), 6.93 (s, 1H), 6.94 (d, *J* = 8.7 Hz, 1H), 6.96 (s, 1H), 7.03 (s, 1H), 7.57 (d, *J* = 8.7 Hz, 1H), 8.17 (s, 1H). ^13^C-NMR (CD_3_OD) δ: 56.4 (CH_3_), 112.6 (CH), 115.4 (CH), 117.3 (CH), 117.4 (CH), 118.8 (C), 121.6 (CH), 125.3 (C), 126.3 (C), 134.1 (C), 147.4 (C), 147.8 (C), 149.2 (C), 171.0 (C), 154.6 (CH), 178.5 (C).

#### 3.1.2. Isoflavone Solubilization

For the pharmacological assays, fresh solutions of TM were prepared daily by dissolving in 30 µL of DMSO (CAQ – Casa da Química Ind. E Com. Ltd, Diadema, SP, Brazil); this volume of solvent did not alter the basal response of the neuromuscular preparation [47,48].

### 3.2. Pharmacological Assays

#### 3.2.1. Venom and Purification of BthTX-I

*Bothrops jararacussu* venom was collected from two adult specimens kept in the Serpentário do Centro de Estudos da Natureza (CEN). The venom was lyophilized and certified by Dr. José Carlos Cogo (Vale do Paraiba University—UNIVAP, São José dos Campos, SP, Brazil). BthTX-I was purified and its identity confirmed as described by Homsi-Brandeburgo *et al.* [11].

#### 3.2.2. Animals

Male Swiss white mice (26–32 g) were supplied by Anilab (Animais de Laboratório, Paulínia, SP, Brazil). The animals were housed at 25 ± 3 °C on a 12 h light/dark cycle and had access to food and water *ad libitum*. This project was approved by the institutional Committee for Ethics in Animal Research of Vale do Paraiba University (protocol no. A013/CEUA/2011) and the experiments were done in accordance with the general guidelines of the Brazilian Society for Laboratory Animal Science (SBCAL).

#### 3.2.3. Mouse Phrenic Nerve-Diaphragm Muscle (PND) Preparation

The PND preparation [49] was obtained from mice anesthetized with halothane (Cristália, Itapira, SP, Brazil) and killed by exsanguination. The diaphragm was removed and mounted under a tension of 5 g/cm in a 5 mL organ bath containing aerated Tyrode solution (control) of the following composition (mM): NaCl 137, KCl 2.7, CaCl_2_ 1.8, MgCl_2_ 0.49, NaH_2_PO_4_ 0.42, NaHCO_3_ 11.9 and glucose 11.1. After equilibration with 95% O_2_/5% CO_2_ (*v*/*v*), the pH of this solution was 7.0. The preparations were indirectly stimulated with supramaximal stimuli (4× threshold, 0.06 Hz, 0.2 ms) delivered from a stimulator (model ESF-15D, Ribeirão Preto, SP, Brazil) to the nerve by bipolar electrodes. Isometric twitch tension was recorded with a force displacement transducer (cat. no. 7003, Ugo Basile, Varese, Italy) coupled to a 2-channel Gemini physiograph recorder (cat. no. 7070, Ugo Basile) via a basic preamplifier (cat. no. 7080, Ugo Basile) [33]. The preparations were allowed to stabilize for at least 20 min before initiating the treatments described below.

Control preparations were incubated with Tyrode solution alone (*n* = 6), whereas other preparations were incubated with TM (200 µg/mL, *n* = 4), venom (40 µg/mL, *n* = 6) or a mixture of TM + venom preincubated for 30 min at 37 °C prior addition to the organ bath (*n* = 4) [33]. BthTX-I (20 µg/mL) was assayed in two protocols: in one, PND preparations were incubated with toxin followed by the addition of TM (200 µg/mL) while in the other the toxin was preincubated with TM (200 µg/mL) prior to testing in PND.

#### 3.2.4. Quantitative Histological Analysis

At the end of the incubations (120 min), the preparations from the various protocols (control, TM, venom and venom + TM) were processed for histological analysis. The preparations (*n* = 3 for each treatment) were fixed in Bouin solution and processed by routine morphological techniques. Cross-sections (5 µm thick) of diaphragm muscle were stained with 0.5% (*w*/*v*) hematoxylin-eosin for examination by light microscopy. Tissue damage (edema, intense myonecrosis characterized by muscle fiber atrophy, a hyaline aspect, sarcolemmal disruption and myofibril lysis) was expressed as a myotoxicity index (MI), defined as (the number of damaged muscle cells/the total number of cells) × 100 in three non-overlapping, non-adjacent areas of each preparation [28].

#### 3.2.5. *In vitro* Mutagenicity Assay

Mutagenic activity was evaluated by the *Salmonella* microsome assay, using the *Salmonella typhimurium* test strains TA98 and TA97a, kindly provided by Dr. B.N. Ames (Berkeley, CA, USA), with (+S9) and without (−S9) metabolization, by the preincubation method [50]. As discussed in the Results section, the OECD [41] recommends the use of five strains (*S. typhimurium* 1535, TA97a, TA98, TA100, TA102), but this was not feasible in this study because of the low yield of isoflavonoid during extraction. The strains were grown from frozen cultures overnight for 12–14 h in Oxoid Nutrient Broth No. 2. The metabolic activation mixture (S9 fraction), prepared from livers of Sprague–Dawley rats treated with the polychlorinated biphenyl mixture Aroclor 1254 (500 mg/kg), was purchased from Molecular Toxicology Inc. (Boone, NC, USA) and freshly prepared before each test. The metabolic activation system consisted of 4% S9 fraction, 1% 0.4 M MgCl_2_, 1% 1.65 M KCl, 0.5% 1 M D-glucose-6-phosphate disodium, 4% 0.1 M NADP (nicotinamide adenine dinucleotide phosphate), 50% 0.2 M phosphate buffer and 39.5% sterile distilled water. The isoflavone was dissolved in DMSO (Sigma Chemical Co., St. Louis, MO, USA) to provide a non-toxic concentration of 0.19 mg/plate. This concentration was added to 0.5 mL of 0.2 M phosphate buffer, or to 0.5 mL of 4% S9 mixture, with 0.1 mL of bacterial culture and then incubated at 37 °C for 20–30 min. Subsequently, 2 mL of top agar [0.6% agar, histidine and biotin (0.5 mM each) and 0.5% NaCl] was added and the mixture poured onto a plate containing minimal agar. The plates were incubated at 37 °C for 48 h and the His+ revertant colonies were counted manually. All experiments were done only in duplicate because of the low amount of isoflavone available. The use of duplicate plating is acceptable when scientifically justified [41]. The results were analyzed with the statistical software package Salanal 1.0 (U.S. Environmental Protection Agency, Monitoring Systems Laboratory, Las Vegas, NV, from Research Triangle Institute, Research Triangle Park, NC, USA), using the model of Bernstein *et al.* [51]. The mutagenic index (MI), defined as the average number of revertants per plate with the test compound divided by the average number of revertants per plate with the negative (solvent) control, was also calculated. A sample was considered positive when the MI was equal to or greater than two for at least one of the concentrations, and if it had a reproducible dose-response curve [52]. The standard mutagen used as a positive control in experiments without the S9 mix was 4-nitro-o-phenylenediamine (NOPD, 10 μg/plate). In experiments with S9 activation, 2-anthramine (2-AA, 1.25 μg/plate) was used. DMSO (50 μL/plate) served as the negative (solvent) control.

#### 3.2.6. Statistical Analysis

Each pharmacological protocol was repeated at least four times and the results are shown as the mean ± SEM. The number of experiments (*n*) is indicated in the corresponding figure legend. Student’s *t*-test was used for statistical comparison of the data and the confidence level was set as 5% (α = 0.05).

## 4. Conclusions

In conclusion, the bioactive isoflavone, 7,8,3'-trihydroxy-4'-methoxyisoflavone (TM) from *D. alata* Vogel efficiently counteracted the myotoxicity and neuromuscular activity of *B. jararacussu* venom and its major myotoxin, BthTX-I, *in vitro*. TM was not mutagenic in *S. typhimurium* strains TA 97a and TA 98, indicating its safety. The results of this study reinforce the potential of TM for therapeutic use in the treatment of venomous snakebites.
